# Sleep-Disordered Breathing: Key Pathophysiological Mechanisms and Their Clinical Implications

**DOI:** 10.3390/life16101658

**Published:** 2026-10-02

**Authors:** Julia Rupp, Emily Heese, Alberto Francisco Monegro

**Affiliations:** Jacobs School of Medicine and Biomedical Sciences, University at Buffalo, Buffalo, NY 14203, USA

**Keywords:** sleep-disordered breathing, obstructive sleep apnea, phenotypes, endotypes, central sleep apnea, obesity hypoventilation syndrome, upper airway resistance syndrome

## Abstract

Sleep-disordered breathing (SDB) encompasses a spectrum of conditions characterized by abnormal respiratory patterns during sleep. These disorders include obstructive sleep apnea (OSA), central sleep apnea (CSA), hypoventilation syndromes, primary snoring, and upper airway resistance syndrome (UARS). These disorders disrupt normal sleep architecture, contributing to sleep fragmentation, impaired daytime function, and significant cardiometabolic morbidity. This review synthesizes current understanding of the physiologic mechanisms underlying SDB, beginning with normal sleep regulation and extending to disease-specific pathophysiology. By integrating pathophysiological mechanisms with their clinical implications, this review provides a comprehensive framework for understanding disease heterogeneity and progression. The goal of this review is to provide clinicians with the resources to inform more precise diagnoses, patient classifications, and treatment strategies for sleep-disordered breathing. OSA is characterized by repetitive upper airway collapse, which can be further characterized by emerging phenotypes and endotypes that highlight heterogeneity in the clinical presentation and polysomnographic features. CSA, in contrast, results from diminished or absent respiratory drive and is frequently associated with heightened chemoreflex sensitivity and ventilatory instability. Obesity hypoventilation syndrome represents a severe form of SDB marked by impaired respiratory mechanics, reduced lung compliance, and blunted ventilatory drive, partly mediated by leptin resistance. Primary snoring and Upper Airway Resistance syndrome exist along a continuum of increasing airway resistance and collapsibility, with emerging evidence suggesting these conditions may not be entirely benign. Additionally, there may be overlaps between diagnoses with patients meeting the criteria for multiple. Classification of SDB has evolved through integration of clinical features, polysomnographic parameters, and pathophysiologic mechanisms.

## 1. Introduction

Sleep disordered breathing is an umbrella term that describes a range of conditions characterized by abnormal respiratory patterns during sleep. These disorders are classified as either obstructive sleep apnea (OSA), central sleep apnea (CSA), hypoventilation with or without hypoxemia, primary snoring, and upper airway resistance syndrome (UARS) [1]. Sleep disordered breathing can result in sleep fragmentation, impacting normal sleep physiology and daily function. The goal of this review is to discuss the mechanisms and pathophysiology of the conditions classified as sleep disordered breathing, with attention toward the clinical implication of viewing sleep disorders as complex and heterogeneous conditions.

## 2. Materials and Methods

The present paper is a narrative review discussing the pathophysiology of sleep disordered breathing. For the purposes of this review, PubMed searches were conducted. The search keywords utilized were Obstructive Sleep Apnea, Primary Snoring, Central Apnea, Upper Airway Resistance Syndrome, Hypoventilation, and Obesity Hypoventilation Syndrome. Initial search resulted in 3619 articles. Articles were initially screened by title and abstract review. Subsequent review of the text and discussion of article content amongst writers was done to determine final inclusion. Articles were included if they focused on mechanisms and/or pathophysiology of sleep disordered breathing. Article types included were clinical trials, meta-analysis, systematic reviews, clinical guidelines, and review papers. Selected articles included data on adult and pediatric population, as well as human and animal studies.

## 3. Normal Sleep Physiology

### 3.1. Neural and Hormonal Regulation of Sleep

#### 3.1.1. Regulation of Sleep

The current understanding of sleep regulation is driven by a two-model process, with a homeostatic process (Process S) and circadian process (Process C). The interaction of Process S and Process C governs the timing and intensity of sleep [2]. During the circadian day, the escalating circadian wake-promoting signal counterbalances accumulating sleep debt, allowing humans to remain awake for the normal 16 h day. Conversely, during the circadian night, the low circadian wakefulness drive coincides with the high homeostatic sleep pressure, making it difficult to stay awake [3].

#### 3.1.2. Homeostatic Drive and Sleep Load

Process S reflects sleep debt and increases during wakefulness and declines during sleep [4]. A transition to sleep or wake occurs when Process S reaches its upper or lower threshold, respectively [2]. Sleep pressure builds as an exponential function during wakefulness and decays at an exponential rate during sleep. Therefore, the longer the duration of wakefulness, the higher the drive to sleep and the deeper and longer the subsequent sleep episode will be. The required duration of sleep varies between species, with humans averaging a need of 7 h of sleep per night [5]. Additionally, Adenosine is a well characterized molecular mediator of homeostatic sleep drive, as it increases in the cortex and basal forebrain during prolonged wakefulness and decreases during the sleep recovery phase [6].

#### 3.1.3. Circadian Rhythm

The circadian rhythm refers to a cycle that is slightly longer than 24 h that regulates many aspects of physiology and behavior in addition to sleep and waking, such as hormone secretion, body temperature, and urine production [3,7]. The central pacemaker that coordinates within cells and between organ systems is the suprachiasmatic nucleus (SCN) in the hypothalamus [8]. Most aspects of sleep are determined by the time at which sleep occurs. However, the circadian system is a major determinant of the timing of sleep and the internal structure of sleep, having the biggest impacts on REM sleep, sleep latency, and sleep consolidation [8].

The sleep-wake propensity of the circadian rhythm is set up such that the strongest drive for wakefulness occurs in the evening, near the end of the usual wake episode. After 1900 h, the circadian wake-promoting signal rapidly decreases, leaving a virtually unopposed homeostatic sleep drive that enables sleep onset [3]. Similarly, the strongest drive for sleep occurs in the very early morning hours, while you are still typically asleep, near the end of a usual sleep episode [7,9].

The hypothalamic-pituitary-adrenal axis is reciprocally coupled to the circadian rhythm. Cortisol nadir coincides with the predominance of SWS and reaches its maximum near morning, when REM sleep dominates. The onset of sleep has an inhibitory effect on cortisol secretion, and awakening is associated with increased levels of cortisol. Additionally, CRH promotes wakefulness and decreases SWS and REM sleep [10]. Dysregulation of this axis has been implemented in conditions of sleep dysregulation [11].

#### 3.1.4. The Role of Melatonin

Melatonin plays a multifaceted role in sleep, serving as a circadian phase shift, sleep facilitating agent, and a thermoregulatory signal [12,13]. Melatonin is synthesized in the pineal gland in the dark phase of the light/dark cycle. The result is that circulating levels of melatonin begin to rise in the evening, peak in the middle of the night, and then decrease to low daytime levels by the end of the night [13]. The initial evening surge of melatonin is known as the dim light melatonin onset and typically occurs 2 h before sleep onset [14]. The dim light melatonin onset is closely correlated with the collapse of the circadian wake-promoting signal that results in a marked increase in desire to sleep [15].

### 3.2. Sleep Cycle

The sleep-wake cycle is divided into three stages: wakefulness, non-rapid eye movement (NREM), and rapid eye movement (REM). During sleep, the NREM and REM stages are organized into recurring cycles lasting approximately 90–120 min. Respiratory control is highly dependent on the sleep phases; therefore, an understanding of normal sleep physiology is essential for discussions on the pathophysiology of sleep disordered breathing [16].

The wakefulness stage is characterized by electroencephalogram (EEG) as a posterior dominant (alpha) rhythm with eye blinks and reading eye movements. NREM sleep is further subtyped into the N1, N2, and N3 phases. The N1 phase is the transitional period between wakefulness and sleep and is characterized on electroencephalogram (EEG) with slow eye movements, theta waves, and vertex sharp waves. The N2 phase is notable for the presence of sleep spindles and K-complexes on EEG and comprises approximately 50% of total sleep. The N3 or “deep sleep” phase is characterized by high-amplitude delta waves on EEG [16]. Slow wave activity (SWA), denoted by high-amplitude, low frequency activity, during NREM sleep is a key electrophysiologic marker of sleep pressure and its intensity is strongly correlated with the previous wake duration [17]. Lastly, REM sleep consists of low-amplitude, mixed frequency activity with sawtooth waves and rapid eye movements seen on EEG [16].

### 3.3. Respiratory Control in Sleep

During wakefulness, control of breathing is determined by chemoreceptor-mediated feedback as well as a wakefulness drive to breathe. This wakefulness stimulus is a tonic excitatory neural input that modulates and stabilizes ventilation independently of chemoreceptors [18]. During sleep, this drive is blunted. Ventilatory control during NREM sleep is driven by the metabolic state as sensed by chemoreceptors [19]. Conversely, REM sleep is partially uncoupled from the control of chemoreceptors and is instead maintained by endogenous brainstem drivers [20].

## 4. Primary Snoring and Upper Airway Resistance Syndrome

**Figure 1:**
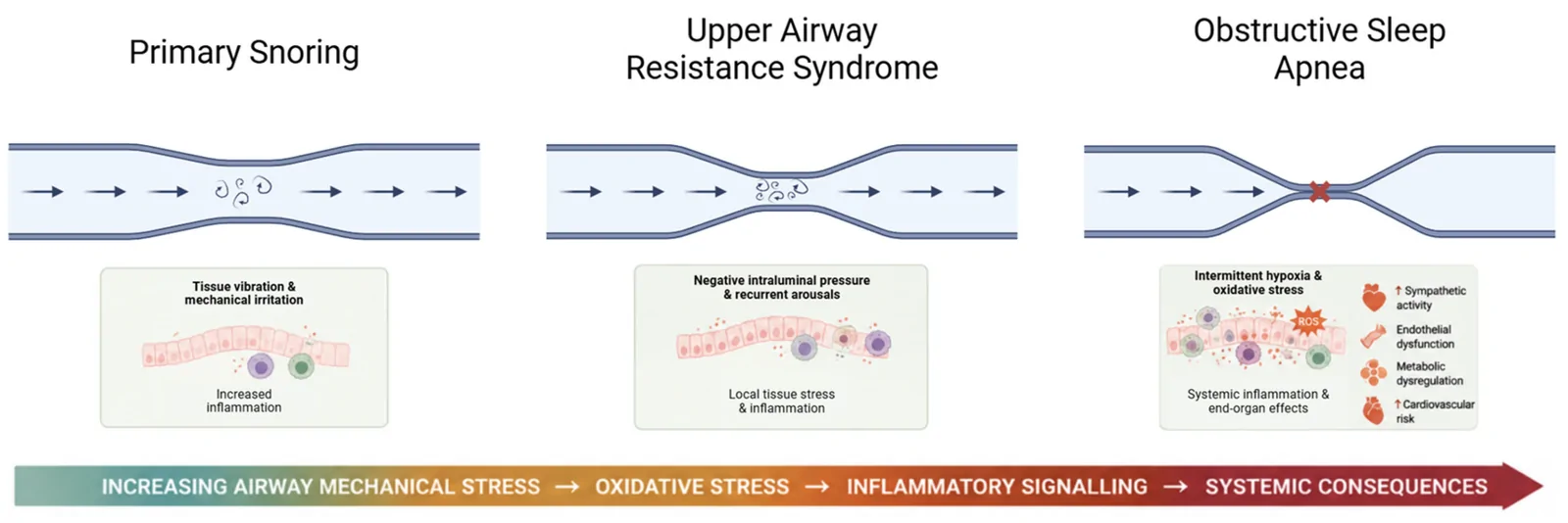
Primary snoring, upper airway resistance syndrome, and obstructive sleep apnea. Venturi tubes which conceptually differentiation the levels of obstruction between primary snoring, UARS, and OSA. The inflammatory consequences of each are described below. Arrows within airways demonstrate airflow.

Primary snoring, also known as simple snoring, is defined as the presence of snoring with fewer than five apneas and/or hypopneas per hour (Table 1) [21]. Snoring is produced by turbulent airflow and tissue vibration of upper airway soft tissue structures. Primary snoring, Upper airway resistance syndrome (UARS), and OSA exist as a continuum of increasing severity, airway collapsibility, and respiratory effort (Figure 1) [22]. Specifically, primary snoring lacks the flow limitations seen in UARS and OSA. UARS is characterized by increased upper airway resistance that produces repeated respiratory-effort–related arousals and sleep fragmentation, despite the absence of the apneas or hypopneas required for a diagnosis of OSA.

The main mechanism driving primary snoring is pharyngeal narrowing upon inspiration, a concept explained by the Bernoulli principle. This principle states that increased velocity of airflow results in a pressure drop at that point. The upper airway is shaped like a Venturi tube such that it starts wide, narrows at the soft palate, and then widens again. The narrow soft palate causes air flow velocity to increase, which decreases pressure and ultimately causes inward displacement of the flexible soft palate [23]. Increasing lengths of the pharyngeal airway further promotes snoring according to Poiseuille’s law, which states that a longer tube results in a greater pressure gradient. During REM sleep specifically, pharyngeal muscle tone decreases, which increase airway compliance and the risk of narrowing [24].

The passive pharyngeal critical closing pressure (Pcrit) defines the pressure at which the upper airway collapses significantly enough to limit airflow and produce an auditory snore. Children have a more negative Pcrit than adults with primary snoring and the most common anatomic location of collapse is at the overlap between the adenoids and tonsils [25].

Once displaced, the soft palate experiences a flow-induced flutter. Aerodynamic forces are delayed behind the tissue motion, which allows a self-excited oscillatory pattern of collapsing and reopening [26]. Fluttering is highly characteristic of primary snoring because it is seen in a patent but narrow airway. Any condition which promotes narrowing and turbulence predisposes to snoring, such as a long soft palate, nasal obstruction, and obesity [27]. A cross-sectional study showed vertical craniofacial growth patterns, such as a long-face morphology was associated with increased respiratory-related mandibular movements that predisposed to unstable breathing patterns [28].

Primary snoring has largely been considered a benign condition; however, recent evidence suggests it may be associated with adverse cardiovascular changes. A 2025 cross sectional study demonstrated that patients with primary snoring had significantly increased carotid intima-media thickness compared to the control group. These findings were not replicated in the femoral artery, which raises the possibility that local mechanical vibrations from snoring may contribute to vascular changes [29]. Supporting this proposed mechanism, Cho et al. exposed the carotid arteries of rabbits to 6 h of direct vibration found endothelial dysfunction, providing mechanistic evidence that local vibration may contribute to vascular injury [30]. Further research is needed to determine the applicability of these findings to humans. Additionally, a meta-analysis found that primary snorers had a 28% higher risk of coronary artery disease, although these observational findings do not establish a causal relationship [31]. In addition to the cardiovascular complications, multiple cross-sectional cohort studies have shown that primary snoring is associated with excessive daytime sleepiness and a decline in cognitive function [32,33]. Pediatric cross-sectional studies by Brockmann et al. provide further compelling evidence as primary snoring has been associated with hyperactive or inattentive behavior and poor school performance [34].

## 5. Obstructive Sleep Apnea

Obstructive sleep apnea (OSA) is a complex disorder with heterogeneity in its presenting symptoms, physiologic etiology, severity, and management (Table 2). The gold standard diagnosis is polysomnography (PSG), with the Apnea-Hypopnea Index (AHI) used in correlation with clinical symptoms. Calculated from the number of apneas and hypopnea events per hour, the AHI alone does not capture the heterogeneity of OSA. Grouping OSA into phenotypes based on symptoms and patient characteristics, as well as endotypes based on the pathophysiology can help identify unique subgroups of patients for targeted treatment [35,36]. This can be done via stratification based on clinical presentation, pathophysiology, and molecular features [35].

### 5.1. Clinical Phenotypes

OSA is a heterogeneous disorder with significant variability in clinical presentations and comorbidities. Historically, OSA was considered a single disorder defined by the frequency of respiratory events during sleep. However, AHI alone does not address the severity of events, type of respiratory disturbance, associated symptoms, or the underlying etiology of OSA. Cluster analyses in large and geographically diverse populations have demonstrated this and highlighted how AHI alone does not fully capture the diversity and heterogeneity of OSA [37,38]. Resultantly, emphasizing the phenotypic and endotypic classifications of OSA has become pivotal in the patient-centered approach to care.

#### 5.1.1. Symptom Based Phenotypes

OSA has a wide range of clinical presentations, from those who experience minimal symptoms to those with significant excessive daytime sleepiness. Two Icelandic studies developed symptomatic clusters of OSA from patients in the Iceland Sleep Apnea Cohort (ISAC) based on previously standardized sleep questionnaires [35,39]. Ye et al. analyzed 822 patients with newly diagnosed moderate-to-severe OSA and identified three distinct clusters: a disturbed sleep group (cluster 1), a minimally symptomatic group (cluster 2), and an excessive daytime sleepiness group (cluster 3), making up 32.7%, 24.7%, and 42.6% of the cohort, respectively [39]. Romero-Peralta et al. conducted a single center and retrospective analysis on a cohort of 2454 patients referred for suspected OSA. In contrast to Ye et al., patients with AHI > 5 were included. They also noted clusters with minimal sleepiness and snoring. In both studies, the “excessive daytime sleepiness” cluster presented with the most “typical” OSA symptoms of nighttime breathing pauses and snoring that disrupted someone else’s sleep [35,39]. The traditional classification modality of AHI did not differ between clusters. However, comorbid hypertension, diabetes, and cardiovascular disease still had a relatively increased prevalence in cluster 2. The pattern persisted at the 2-year ISAC follow-up: the minimally symptomatic group had higher rates of hypertension (55.0% vs. 46.7% and 41.6%; *p* = 0.020) and cardiovascular disease [40].

In the Sleep Apnea Global Interdisciplinary Consortium (SAGIC), 972 patients with moderate-to-severe OSA were found to have clusters of symptoms defined by disturbed sleep, minimally symptomatic, and excessively sleepy clusters, in addition to other subtypes that were characterized by upper airway symptoms or moderate sleepiness [35]. Similar subtypes were found in population-based Korean Genome and Epidemiology Study (KoGES), a Chilean clinical cohort study by Labarca G, et al., and in a large study by from the Pays de la Loire cohort in France, which included 5358 patients with OSA and no overt cardiovascular disease [41,42,43].

In the Sleep Heart Health Study (SHHS), four subtypes were identified among 1207 participants with AHI ≥ 15: disturbed sleep (12.2%), minimally symptomatic (32.6%), excessively sleepy (16.7%), and moderately sleepy (38.5%). Compared with individuals without OSA, the excessively sleepy subtype had a significantly greater risk of incident cardiovascular disease (HR 2.01; 95% CI 1.25–3.23). That subtype also carried more than three times the risk of prevalent heart failure compared with the other subtypes [44]. In Labarca G, et al., patients with excessive sleepiness had an increased risk of incident cardiovascular mortality [42].

The literature suggests that both minimally symptomatic phenotypes and excessive sleepiness phenotypes can carry a burden of hypertension and cardiovascular disease. However, randomized trials that enrolled predominantly patients without excessive sleepiness did not show a cardiovascular benefit of CPAP in their primary analyses [45,46,47]. Minimally symptomatic patients from ISAC reported significant decreases in daytime sleepiness and physical fatigue after treatment, with larger improvements among PAP users [40]. This suggests that “minimal symptoms” may partly reflect adaptation to chronic, unrecognized impairment.

#### 5.1.2. Gender Differences in Sleep Phenotypes

Patients with OSA symptoms can present with complaints of difficulty sleeping, disturbed sleep, or insomnia. This phenotype has been observed predominantly in women. OSA has a relatively high prevalence across genders, with a mean prevalence of 22.5% in women and 27.5% in men [48,49]. Despite the high prevalence in women as well, most cluster analysis research has been done with predominantly male populations. Recent research, however, has shown that OSA may exhibit distinct characteristics in women [49,50,51].

Females with OSA are less likely to exhibit snoring and witnessed apneic episodes, both of which are more likely in males with OSA [50]. Symptoms of daytime fatigue, lack of energy, insomnia, mood disturbances, and morning headaches affect females at a higher rate than male counterparts [51]. Additionally, at similar AHI levels, females tend to report more impairment in daily function, including impaired work performance, decreased quality of life, and increased sick leave [52,53,54]. During sleep itself, OSA is still distinct in female patients. Shorter apneic episodes and clustering of apneic episodes during REM is present during sleep in females with OSA, whereas males have a more uniform spread of apneic episodes throughout REM and NREM sleep [55]. Since NREM accounts for most of the time in sleep, this may contribute to an increase in diagnosis and prevalence of OSA in males. Furthermore, supine position worsens OSA to a lesser extent than in males, and oftentimes nighttime respiratory events may not meet criteria for true apneic events or upper airway obstruction [50,51]. Anatomical and ventilatory control responses make the female upper airway less collapsible and more responsive to obstruction, which helps mitigate breathing instability and prevent complete obstruction [51]. However, females experience prolonged partial upper airway obstruction and increased pCO_2_ during sleep. This still causes increased work of breathing and recent arousals that lead to non-restorative sleep and daytime fatigue, evidenced by CPAP treatment resulting in higher attentiveness and decreased daytime sleepiness [51,56].

To evaluate how the difference in clinical presentation in female OSA affected phenotyping, Fontanilles Arbones et al. performed a cluster analysis with an exclusively female OSA population [57]. They defined four new phenotypes, none of which have been described in male OSA cluster analyses before. Interestingly, the “classic phenotype”, defined as daytime sleepiness with cardiovascular comorbidities, was the most common amongst this female cohort, followed by the “atypical phenotype” with mood disorders and non-restorative sleep [57]. Further cluster analysis of female OSA is necessary to increase awareness of clinical presentations, ensure adequate testing and diagnosis, and guide prognostic and therapeutic modalities.

### 5.2. Polysomnographic and Physiological Phenotypes

Despite being a routine test in the diagnosis of OSA, polysomnographic features do not yet define OSA subtypes on a universal scale. However, cluster analyses have identified multiple different OSA types. This extends the classification beyond the traditional AHI criteria, accounting for heterogeneity in pathophysiology, symptoms, and comorbidity risk. Based on recent studies, most groups can fit into a hypoxia dominant, arousal dominant, Periodic Limb Movement of Sleep (PLMS)/poor sleep, or hypopnea predominant group.

#### 5.2.1. Hypoxia and Hypopnea Dominant

Hypoxia dominant clusters are defined as substantial nocturnal oxygenation impairment as characterized by prolonged saturation less than 90%. The hypopnea-predominant phenotype is defined as a high proportion of hypopneas relative to apneas [1].

Hypopneas occur when there is dynamic airway obstruction and these episodes occur in abundance particularly in very obese patients as there are pathophysiologic measures working against complete upper airway collapse [58]. In a 2023 cross-sectional analysis by Zinchuk et al., two groups with significant hypoxia during sleep were identified, described as the ‘REM and hypoxia’ and ‘hypopnea and hypoxia’ clusters. ‘Hypopnea and hypoxia’ spent 14% of sleep time at less than 90% oxygen saturation, which is the highest of the clusters. However, only ‘hypopnea and hypoxia’ had a significantly higher association with ACS, TIA, stroke, or death compared to the ‘mild OSA’ group [59]. Similarly, a cluster analysis in Korea showed that the ‘hypoxia’ cluster had a significantly higher disease mortality risk (HR = 7.6) and all-cause mortality compared to the mild OSA cluster compared to mild OSA [60]. Additionally, patients with this phenotype are more likely to have dynamic obstructions, causing flow limitation without complete airway closure. Patients also tend to be older and include more women [61]. Surgical treatment, such as maxillomandibular advancement, has a significantly higher cure rate in hypopnea predominant versus apnea predominant phenotypes. Post-operative evaluation showed marked improvements in AHI, apnea index, hypopnea index, and oxygen desaturation index [62].

Hypoxia predominant OSA is important to consider clinically. Two recent observational studies by Labarca et al. and Peker et al. have reported hypoxic burden as an emerging metric to predict cardiovascular disease-related morbidity and mortality. AHI alone did not produce a similar prediction of cardiovascular outcomes [63,64]. Since hypoxic burden shows a cumulative depth and duration of respiratory events, reported in %min/hour, it may better represent severity of hypoxic events. This is compared to the currently used measure of AHI, which detects frequency of apneas and hypopnea, but not the severity. Given its advantage of assessing severity of respiratory events, hypoxic burden remains a promising metric for evaluation of OSA alongside AHI. However, additional research is needed to create standardizations of measurement and to prospectively assess hypoxic burden guided OSA treatment on cardiovascular outcomes [65].

A similar cardiovascular effect was also found for REM predominant OSA. As mentioned above in the study by Zinchuk et al., respiratory events that predominantly occurred during REM sleep are associated with hypoxia [59]. Patients with REM predominant OSA experience significant oxygen desaturation, as REM-related muscle atonia contributes to increased airway collapsibility [66].

#### 5.2.2. Apnea Predominant

**Figure 2:**
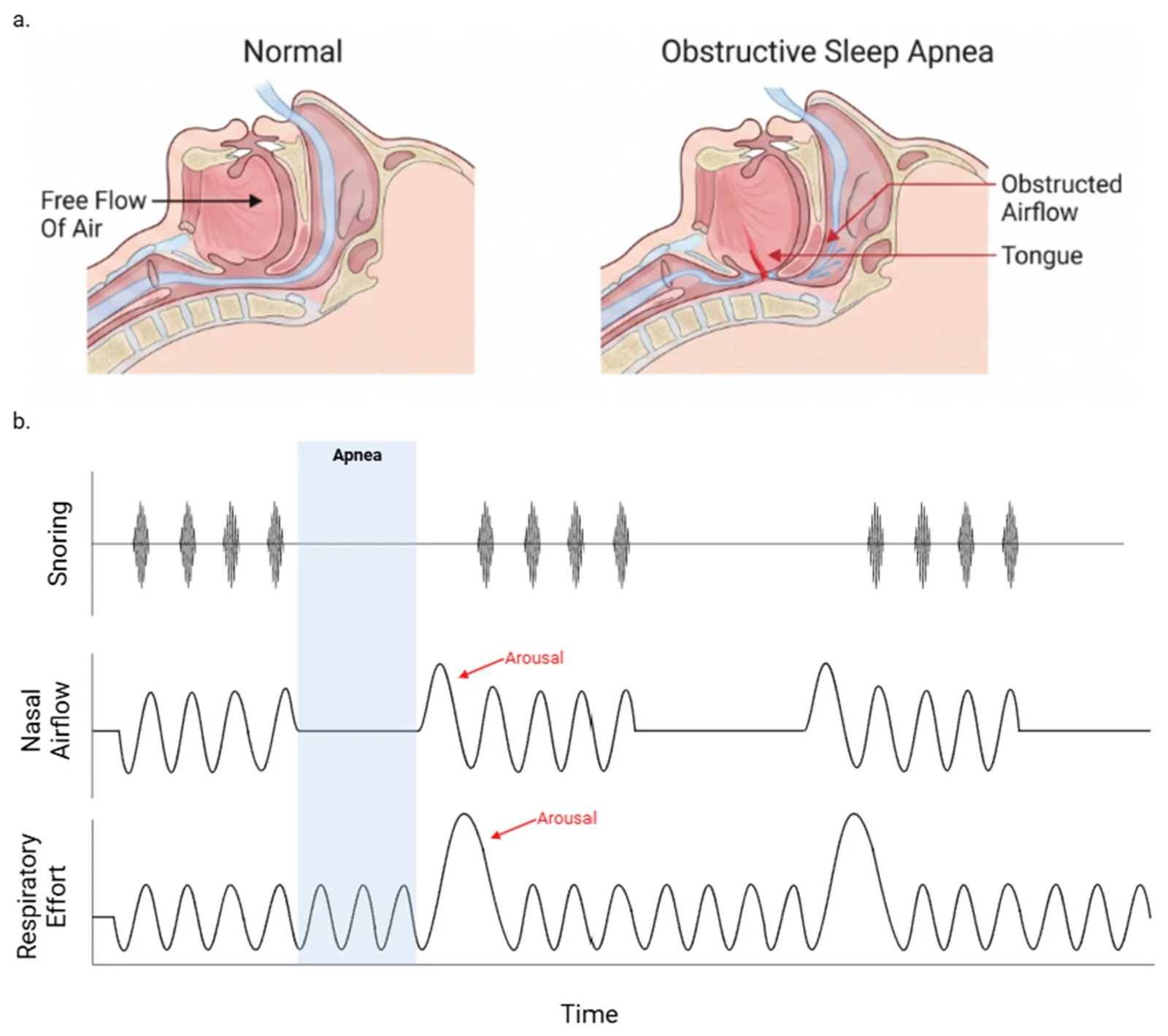
Upper airway obstruction and respiratory consequences of obstructive sleep apnea. (**a**) Comparison of a normal, patent airway (**left**) with an obstructed airway (**right**) showing upper airway collapse in which a posterior displacement of the tongue limits airflow. (**b**) Schematic respiratory tracing demonstrating recurrent obstructive apneas with cessation of nasal airflow (**bottom**), normal respiratory effort followed by an arousal which concludes the apnea (**middle**), and an example snoring pattern (**top**).

Apnea predominant OSA is considered in patients whose AHI is greater than 50% obstructive apneas [67]. While apneas cause less frequent arousals in NREM sleep stages, the arousals that are triggered are longer in duration than those in the hypopnea phenotype [68]. The desaturations associated with apnea predominant OSA are also more severe than hypopnea predominant [68]. Static obstruction predominates in the apnea predominant phenotype, during which patients have complete airway collapse with no airflow despite ongoing respiratory efforts, as demonstrated in Figure 2 [69]. Hypertension and diabetes are common comorbidities in the apnea predominant phenotype [70]. However, the cardiovascular risk within phenotypes is variable across studies as the hypopnea predominant phenotype carries increased risk of cardiovascular comorbidities [70].

#### 5.2.3. Low Respiratory Arousal Threshold with Predominant Respiratory Effort Related Arousal

Low respiratory arousal threshold refers to the tendency to awaken too early in response to minor respiratory stimuli. With this interruption of sleep, there is no accumulation of the respiratory drive needed to recruit upper airway dilator muscles and restore stable breathing [61]. In normal compensatory physiology, obstructive events increase CO_2_ and negative intrathoracic pressure, which subsequently activate pharyngeal dilators without cortical arousal. However, in patients with low arousal threshold, premature awakening occurs before air flow or oxygen saturation have decreased enough to classify the event as true apnea or hypopnea. This process perpetuates a fragmented sleep cycle and recurrent obstruction. With each premature awakening, there is compensatory hyperventilation that lowers the CO_2_ below apneic threshold. When sleep begins again, low CO_2_ decreases respiratory drive and pharyngeal muscle tone, which combine to promote upper airway narrowing, thus continuing the cycle [71].

The result of the above process is a respiratory effort related arousal (RERA), a series of flow limited breaths terminated by arousal, but not meeting criteria for apnea or hypopnea [72]. On a polysomnography, a RERA is scored if there is a sequence of breaths lasting at least 10 s characterized by increasing respiratory effort or flattening of the inspiratory portion of the waveform that does not meet the criteria for apnea (>90% airflow reduction) or hypopnea [72]. Patients with OSA who have exclusively RERAs were previously classified as upper airway resistance syndrome, but it is now grouped as a subtype of OSA given the shared pathophysiology and response to treatment [73].

RERAs are excellent markers to assess the neurophysiologic burden of respiratory compromise during sleep on the quality of life of patients. Non-desaturating breathing events are independently associated with objective daytime sleepiness, as measured by the multiple sleep latency test, but less associated with subjective daytime sleepiness, measured by the Epworth Sleepiness Scale [74]. Therefore, patients presenting with non-specific symptoms of OSA, such as daytime sleepiness, fatigue, insomnia, or neurocognitive symptoms, should be evaluated for RERAs. Additionally, RERAs should be included in evaluation if the respiratory disturbance index is greater than 5 and PAP therapy should be considered [73]. RERAs are correlated with AHI severity, emphasizing the importance of evaluating RERAs in potential OSA diagnoses [75]. Clinically, the RERA phenotype is most likely to display daytime sleepiness, neurocognitive symptoms, insomnia, headaches, and GERD [73]. These symptoms mirror the “atypical phenotype” in women OSA that was previously discussed.

#### 5.2.4. Periodic Limb Movements of Sleep/Poor Sleep

Periodic limb movements of sleep (PLMS) in OSA creates a distinct phenotype because these patients are subject to lower arousal thresholds and generally poorer sleep efficiency [76]. Furthermore, PLMS with OSA demonstrated a significantly lower arousal threshold than OSA alone, as well as tendencies towards longer sleep and REM latencies [76,77]. Despite having more difficulty initiating and maintaining sleep and reduced sleep quality on polysomnography (PSG), reported subjective and objective daytime sleepiness did not differ between patients with PLMS and OSA versus OSA alone [76].

Interestingly, patients with PLMS had better hypoxia and apnea profiles than OSA alone. Patients had less proportion of time during sleep at oxygen saturation < 90%, decreased hypoxic burden, and shorter apnea durations, all of which were significant decreases compared to the OSA alone group [77]. Additionally, patients with PLMS and OSA had better upper airway collapsibility profiles and more effective upper airway compensation [77]. PLMS is associated with preserved muscle tone and elevated sympathetic activity during sleep, which may contribute to the better maintained upper airway architecture and decreased hypoxic burden in these patients [78].

### 5.3. Metabolic and Inflammatory Phenotypes

**Figure 3:**
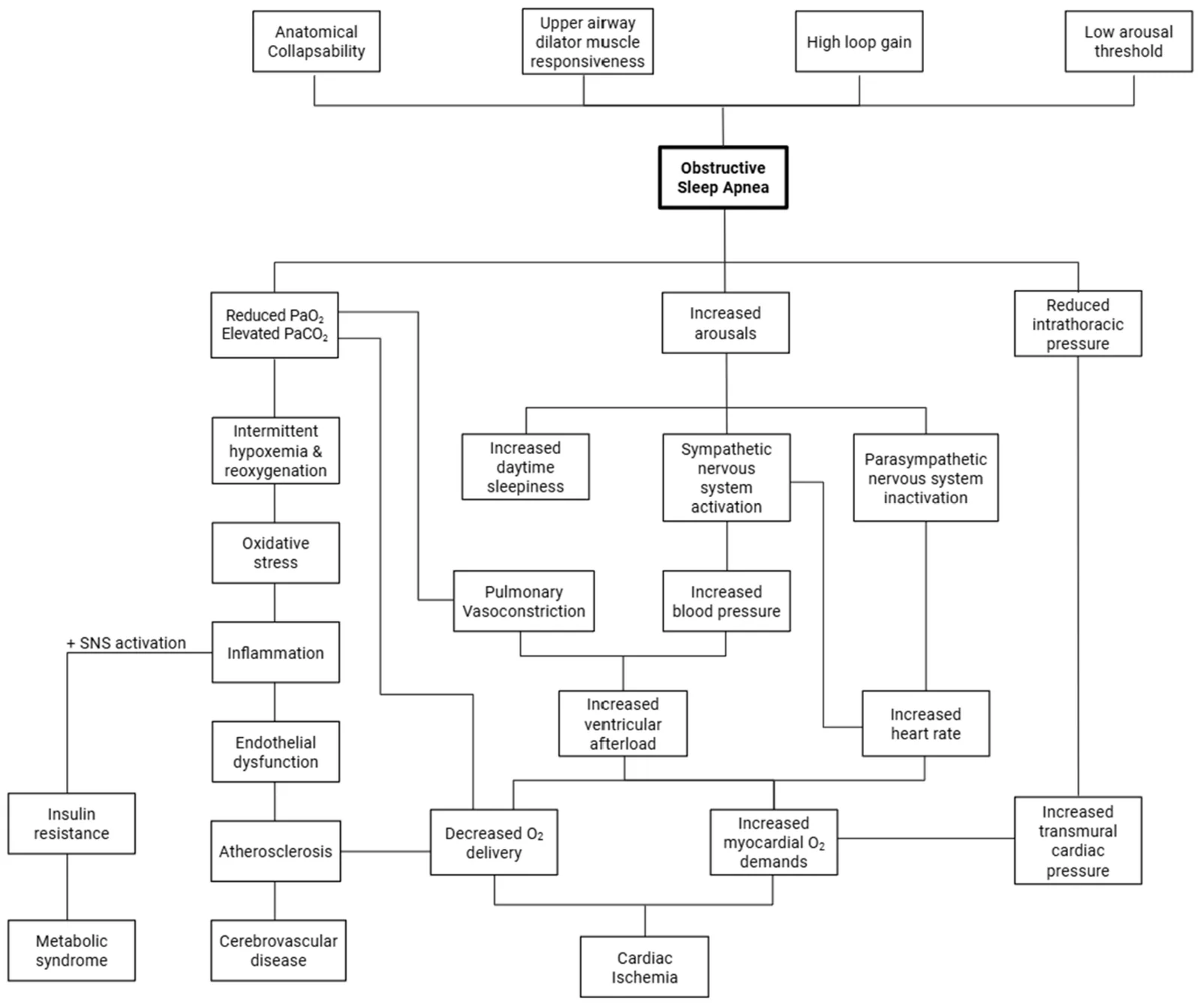
Primary causes and subsequent complications of obstructive sleep apnea. Demonstrates the endotypes of OSA and the mechanisms by which OSA contributes to downstream complications.

The relationship between metabolic and inflammatory processes in OSA is bidirectional, as derangements can both contribute to and result from the comorbidities associated with OSA (Figure 3). The cyclic nature of apneic events produces chronic intermittent hypoxemia (CIH). This cycle of hypoxia and reoxygenation drives the formation of reactive oxygen species, which in turn activate the pro-inflammatory NF-κB cascade [79]. The resultant release of inflammatory cytokines promotes endothelial dysfunction and leukocyte adhesion. The Multi-Ethnic Study of Atherosclerosis found that severe OSA had an independently elevated incident of type 2 diabetes mellitus (T2DM) and worsened insulin resistance [80]. Animal studies proposed the mechanism that CIH further induces a M1-polarized pro-inflammatory phenotype in visceral adipose tissue that causes insulin resistance with impaired glucose metabolism [81]. A randomized control trial studying the effect of the GLP-1 Tirzepatide in adults with moderate-to-severe OSA and obesity showed substantial reductions in AHI and hypoxic burden through the loss of weight [82]. A prospective clinicopathological study by Feng et al. also showed that patients with OSA had higher rates of metabolic dysfunction-associated steatotic liver disease (MASLD), with a higher AHI correlating to increased disease severity [83]. The mechanism driving the liver disease is proposed to involve a CIH-mediated increase in lipogenesis and impairment of fatty acid oxidation.

The sympathetic nervous system (SNS) is frequently overactive in OSA, which contributes to further clinical implications. Frequent arousals, chemoreflex activation following hypoxemia and hypercapnia, and chronic inflammation all contribute to persistent SNS activation. This contributes to resistant hypertension, increased heart rate, increased myocardial oxygen demands, and arrhythmias [84]. Additionally, inspiration against an occluded airway generates markedly reduced intrathoracic pressure. This raises cardiac transmural pressures and increases left ventricular afterload, which produces cardiac strain [84]. Intermittent hypoxemia and hypercapnia during OSA further promote pulmonary vasoconstriction, increasing right ventricular afterload. The Wisconsin Sleep Cohort study found that severe, untreated OSA is linked to a three-fold increase in all-cause and cardiovascular mortality risk [85]. Additionally, a two-fold increase in REM-AHI was associated with 24% higher odds of prevalent hypertension independent of other variables [85]. The Sleep Heart Health Study, a prospective cohort study of over 5000 participants, had similar findings; specifically, that men in the highest obstructive AHI quartile had an adjusted hazard ratio for ischemic stroke of 2.86 [86]. Additionally, a cohort study by Xu et al. demonstrated that hypoxic burden, not AHI, was the main driver of cardiovascular risk [87].

### 5.4. Mechanistic Endotypes of OSA

Obstructive sleep apnea is characterized by partial or complete collapse of the upper airway during sleep. As a result, there is periodic reduction or cessation of air flow, characterized by hypopneas or apneas on polysomnography, respectfully. This is caused by an imbalance of collapsing forces and compensatory mechanisms that keep the airway open [88].

#### 5.4.1. Upper Airway Collapse

Upper airway anatomical abnormalities are a foundation in the pathogenesis of OSA as shown in Figure 2. Patients have varying degrees of upper airway stenosis and collapse, caused primarily by either abnormal maxillofacial structure or soft tissue hyperplasia [89]. OSA has a strong correlation with obesity, which contributes to the common mechanism of upper airway narrowing due to fat deposition in parapharyngeal muscles and fat pads [88,89]. Furthermore, patients with comorbid heart failure, renal failure, or venous insufficiency may experience a fluid shift overnight during which fluid volume moves from the leg to the neck [89]. This can worsen the degree of upper airway collapse. Since as many as 38–40% of individuals with moderate to severe OSA have comorbid heart failure and 30% have comorbid renal failure, it is important to consider how these comorbid conditions can worsen OSA [90,91].

#### 5.4.2. Anatomical Abnormalities

Morphological abnormalities are vital factors in the development of OSA [89]. In children, maxillomandibular defects and adenotonsillar hypertrophy are the most common [92]. Adenotonsillar hypertrophy results in an anatomically narrowed airway. Children have the highest amount of lymphoid tissue around their early school years, resulting in the most adenotonsillar hypertrophy relative to airway size. Around this time at 3–8 years old, there is a peak in pediatric OSA [92,93]. During sleep, muscle atonia and airway collapse occurs in this already narrowed airway, resulting in partial or complete obstruction [92].

Congenital anomalies resulting in airway abnormalities should also be considered. Craniosynostosis, the premature fusion of one of more sutures in the skull, affects an estimated 1 in 2500 births, most associated with syndromes such as Pfeiffer, Apert, and Crouzon [94]. The incidence of OSA in this population varies in the literature but remains relatively high between 50–87% across studies [95,96,97]. The primary contributing factor to OSA development in children with craniosynostosis is midface hypoplasia [94].

In the adult population, inferior displacement of the hyoid bone, increased anterior facial heights, and posterior displacement of the maxilla are common in OSA and result in subsequent oral cavity crowding [98]. Furthermore, enlargement of the soft tissue structures, such as the soft palate and tongue as well as the pharyngeal wall, can invade the airway from the anteroposterior and lateral directions, respectively [99]. Fat deposition in the pharynx and thoracic is one of the major mechanisms of airway compression. Obesity plays a major role in increasing the volume of fat depositions, thus explaining why obesity is such a large risk factor for OSA.

#### 5.4.3. Passive Airway Collapse and Upper Airway Dilator Dysfunction

The genioglossus is the most important upper airway dilator. With each inspiration, the genioglossus contracts and is assisted by the levator and tensor palatini, geniohyoid, and stylopharyngeus muscle to prevent posterior collapse of the tongue [88]. During wakefulness, the dilator muscles maintain pharyngeal patency through coordinated reflex activity. During sleep, however, the coordinated activity and chemical reflex response of the pharyngeal dilator muscles is impaired, thereby increasing the risk of airway collapse [100]. Most pharyngeal dilator muscles lose tone throughout the entirety of sleep, but the genioglossus is unique in that its tone does increase after the onset of sleep, particularly during apneas and hypopneas. A 2012 study observing spontaneous breathing in OSA patients under mild propofol showed that even large increases in genioglossus activity did not increase pharyngeal diameter or inspiratory airflow [101]. The same group of researchers subsequently found that worsening degrees of airway obstruction in OSA patients were associated with increased tone of the genioglossus during sleep. During sleep, airflow limitations and inspiratory airflow limitations caused a greater than twofold increase in genioglossus activation compared to awake measurements. However, activity of the tongue retractors (styloglossus and hyoglossus) were less than half of that measured during wakefulness [101]. These findings combined suggest that other pharyngeal dilator muscles, in coordinated efforts with the genioglossus, are necessary in maintaining airway patency during sleep.

The abnormal susceptibility to pharyngeal collapse in patients with OSA was demonstrated by studying the critical closing pressure (Pcrit). Pcrit is the gold standard to measure pharyngeal collapsibility and it is defined as the pressure at which the airway collapses [102]. Pcrit is derived from the Starling resistor model of the pharynx, which states that pressure gradient drives flow and that flow level is fixed. When the pressure surrounding the pharynx is higher than the internal pressure, the pharynx will collapse. Right before the moment of collapse, the pressure inside the tube is equal to the surrounding pressure; this is the Pcrit [102].

Healthy individuals have a negative Pcrit of about −13 cmH_2_O, which allows the upper airway to remain open [103]. In patients with OSA, the Pcrit becomes less negative and even reaches positive values, depending on the position of the patient. These Pcrit values range from −5 cmH_2_O to 2.5 cmH_2_O across studies, with Pcrit greater than +2 cmH_2_O found in patients with severe OSA [102,103,104]. As the Pcrit value increases, the pharynx becomes more collapsible and there is increased risk of occlusion during sleep, as seen in OSA patients.

#### 5.4.4. High Loop Gain

Loop gain is a measure of the stability of our ventilatory control system. It is a ratio of the ventilatory response to ventilatory disturbance. When breathing is reduced, such as during hypopnea, the CO_2_ levels will rise, triggering a compensatory increase in ventilation. If the ventilatory response is equal to that of the disturbance, blood gases will return to eupneic levels and equilibrium will be re-established. However, if the ventilatory response is unstable, the system will overshoot, causing PaCO_2_ levels to fall below equilibrium. The hypocapnia triggers hypoventilation, upper airway muscle hypotonia, and another airway obstruction, which triggers the cycle to start again. In summary, a large loop gain ratio indicates an unstable respiratory system that is prone to oscillations and leads to excessive ventilation changes in response to small CO_2_ changes [105].

Loop gain can be separated into a controller and plant component, with the controller sensing the stimulus and dictating the outcome of the plant. Chemoreceptor sensitivity to blood gases represents the controller gain and the lung’s ability to alter blood gases represents the plant gain. The product of the two is the loop gain [105]. Chemoreflex responses to hypercapnia and hypoxia decrease as the sleep phases progress, meaning controller gain decreases from NREM to REM sleep [106,107]. Landry et al. evaluated loop gain itself, with data suggesting a decrease in loop gain during REM but a modest increase in loop gain over the course of sleep [108]. This was an interesting finding because the severity of OSA tends to increase during REM sleep, as evidenced by increased AHI scores during REM sleep, which would suggest a higher, rather than lower, loop gain [100,109]. Rather, REM specific physiologic changes of reduced responsiveness of the genioglossus muscle to negative pressure and increased upper airway collapsibility are stronger determinants of respiratory events during REM sleep [108,110].

Recognizing high loop gain as a pathophysiologic contributor to a patient’s OSA has implications for treatment. Oxygen therapy directly affects the carotid body and is considered a therapy aid for OSA due to high loop gain [111]. A 2026 multicenter randomized trial investigated the use of CPAP versus oxygen as therapy for high loop gain OSA. They found that patients treated with CPAP who had improvement in high loop gain events demonstrated a reduction in systolic blood pressure. There was also a correlation between baseline AHI and diastolic BP and CPAP efficacy in improving high loop gain event [111].

### 5.5. Phenotype- and Endotype-Specific Treatment Considerations

Currently, CPAP is the cornerstone treatment for OSA. Some randomized controlled trials have investigated the efficacy of CPAP use in patients with minimally symptomatic OSA for improvement in objective and subjective sleep measures. In 2012, Craig et al. conducted a multicenter, randomized controlled, parallel, hospital-based trial across the UK and Canada with patients who had confirmed OSA with insufficient symptoms to warrant CPAP therapy. In these patients, both subjective sleepiness, measured by the Epworth Sleepiness Scale, and objective sleepiness, measured by Oxford Sleep Resistance, improved with CPAP; however, CPAP did not improve the 5-year calculated cardiovascular risk or any individual components, including blood pressure [112]. Similarly, the MERGE trial done by Wimms et al. in 2020 investigated if 3 months of CPAP plus standard of care improves quality of life in mild OSA [113]. Quality of life, measured by the SF-36 vitality score, significantly increased with CPAP use at 3 months. Secondary outcomes of sleep quality and fatigue were measured by the Epworth Sleepiness Scale, Fatigue Severity Scale, and Insomnia severity index, reporting significantly reduced fatigue and insomnia among those in the CPAP group [113]. Based on the results of these trials, it is evident that even in mild OSA, where patient’s symptoms may seem to not warrant the use of CPAP, therapy significantly improves sleep and quality of life. This emphasizes how critical it is to identify patients with OSA, even without obvious symptom presentation, given the benefit of CPAP therapy.

Standard CPAP adherence is ≥4 h per night, but REM is concentrated in the latter half of sleep. Therefore, patients with REM-predominant OSA who wear CPAP for only the first 4 h of sleep are likely untreated during a significant number of obstructive events. The RICCADSA trial looking at OSA patients with revascularized coronary artery disease found that no significant difference in the intent-to-treat analysis for cardiovascular events. However, a sub-analysis of patients who adhered to CPAP ≥ 4 h per night did experience a reduction in cardiovascular events. While this is a non-randomized correlation, it suggests that adequate CPAP adherence may be necessary to achieve cardiovascular benefit [46]. As clinicians, educating patients on the risk of not using CPAP during the latter half of sleep is essential in ensuring that patients get adequate treatment to prevent adverse cardiovascular outcomes. Other randomized controlled trials, such as the SAVE and ISAACC trials, have not demonstrated a reduction in cardiovascular disease; however, this may be due to inadequate compliance (average use time < 4 h) or heterogenous patient selection [45,47]. A large portion of the patient population in SAVE and ISAACC were of the non-sleepy phenotypic cluster, resulting in notoriously low compliance.

Some specific clinical subgroups have been shown to uniquely benefit from CPAP therapy. In patients with a high hypoxic burden, post-hoc analyses across multiple trials suggest that CPAP may reduce cardiovascular events, with reported relative risk reductions between 17–24% [114]. Similarly, younger patients (<60-years-old) with a high body mass index, high baseline cardiovascular risk, and severe OSA were found in a cohort study to have a 51% lower hazard of major adverse cardiovascular events with regular CPAP use [87]. A different randomized control trial looking at patients with resistant hypertension and OSA (HIPARCO trial) found a clinical meaningful reduction in 24-h ambulatory blood pressure with CPAP use [115]. Finally, a recent meta-analysis of 11 randomized controlled trials showed that greater nightly CPAP use was associated with greater reductions in HbA1c in patients with OSA and T2DM [116].

### 5.6. Oxygen Therapy in OSA

While CPAP treatment of OSA has been shown to reduce systolic blood pressure, the cardiovascular effects of oxygen therapy on OSA are less clear-cut. Ni et al. did not report improvements in systolic blood pressure in a 2026 secondary analysis of the HeartBEAT study [111]. These results differed from a 2019 randomized study by Turnbull et al. that tested a similar hypothesis with higher flow rates of oxygen, finding that supplemental oxygen significantly decreased morning blood pressure [117]. Similarly, Edwards et al. conducted a multicenter randomized crossover trial assessing the effects of either mandibular advancement device, oxygen therapy at 4 L/min, or a combination. Combination treatment yielded significant AHI reduction compared to single treatment mechanisms, as well as non-significant improvements in arousal index and subjective sleep quality [118].

Supplemental oxygen is not currently in the clinical guidelines for treatment of OSA with high loop gain. Further prospective randomized studies and evaluation of clinical outcomes over time would be needed to transform this treatment from investigational to an evidence-based guideline. However, it is clear that phenotypic specific treatments are beneficial to the patient. Physicians should understand the subtype of OSA that they are treating and use that to guide treatment, as it may improve not only symptoms, but cardiovascular risk in the long term.

## 6. Central Sleep Apnea

Central sleep apnea (CSA) is a heterogeneous group of sleep-related breathing disorders characterized by episodes of absent or severely diminished respiratory effort (Table 3). CSA is defined as five or more episodes of central apneas and/or hypopneas lasting greater than ten seconds per hour of sleep with central events compromising greater than 50% of the number of total number of respiratory events [1]. A central apnea is defined as ≥90% drop in airflow from baseline that lasts ≥ 10 s and lacks inspiratory effort characterized by absent thoracoabdominal movements [16].

To fully appreciate the pathophysiology driving CSA, it is important to first consider the neuronal process driving rhythmogenesis. Within the ventrolateral medulla of the brain stem lies the pre-Bötzinger complex (pre-BötC), which has been described as the “kernel structure” essential for inspiratory drive. It contains both excitatory glutamatergic neurons as well as inhibitory GABAergic and glycinergic neurons. Another key player, the retrotrapezoid nucleus (RTN), functions as the primary central respiratory chemoreceptor. This structure detects changes in pH and CO_2_ of the cerebrospinal fluid and has glutamatergic neuronal projections to the pre-BötC. Peripherally, the carotid bodies act as the primary chemoreceptors and possess the ability to sense hypoxemia, hypercapnia, and acidosis. These bodies send signals to the nucleus tractus solitarius (NTS) which can be integrated with central chemoreceptor signals [119,120].

Regardless of the etiology, the severity of CSA increases in the supine position due to the reductions in lung volumes that increase loop gain [121]. CSA is also more common during NREM sleep when the brainstem pattern generator is intrinsically rhythmic, which renders ventilation highly dependent on chemical signaling [122]. Finally, the frequency and duration of CSA events tends to increase later in the night, reflecting progressive elevations in loop gain [123].

### 6.1. Central Sleep Apnea with Cheyne-Stokes Breathing

**Figure 4:**
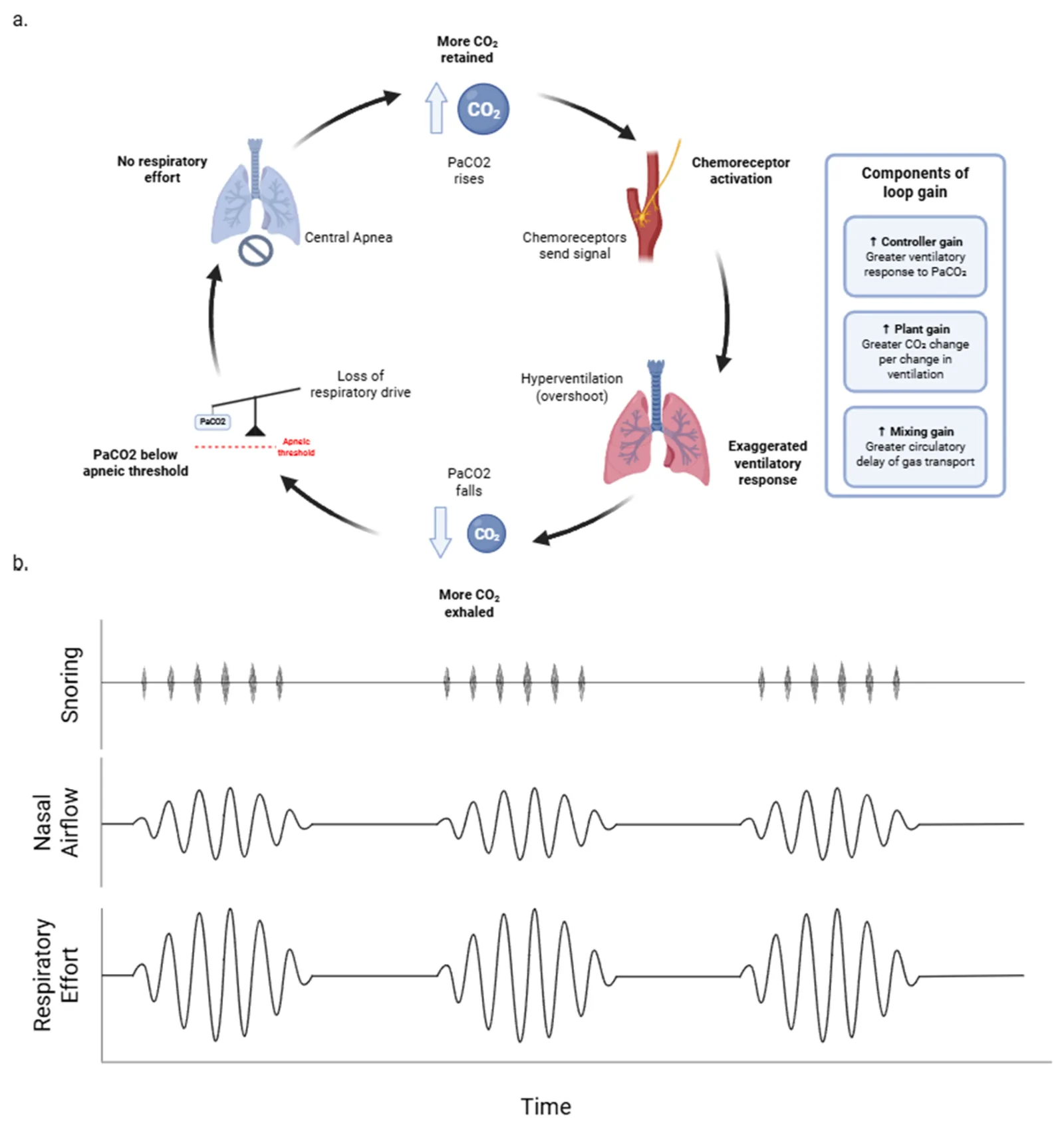
Elevated loop gain and respiratory consequences of central sleep apnea. (**a**) Demonstration of the consequence of elevated loop gain in CSA with CSR. Arrows represent transitions throughout the cycle. (**b**) Schematic respiratory tracing demonstrating recurrent central apneas with cessation of nasal airflow (**bottom**), absent respiratory effort followed with a crescendo-decrescendo pattern (**middle**), and the expected lack of snoring (**top**).

Cheyne-Stokes respirations (CSR) is a form of periodic breathing characterized by a crescendo-decrescendo pattern of ventilation alternating with episodes of apneas. It is defined as ≥3 central apneas and/or hypopneas with a crescendo-decrescendo breathing pattern in a cycle that lasts ≥ 40 s [16]. This is demonstrated in Figure 4.

The engineering term “loop gain” describes the magnitude of response a system has to a disturbance and explains the cyclical mechanisms of CSR (Figure 4). Loop gain reflects the stability of the ventilatory control system and is the product of controller gain (chemoreflex sensitivity), plant gain (how ventilation alters PaCO_2_), and mixing gain (the dynamics of gas transport and circulatory delay). As such, a system with a high loop gain overcorrects and oscillates, manifesting as CSR [19].

Following a brief cessation of breathing, there is a decrease in PaO_2_ and an increase in PaCO_2_ [124]. Peripheral and central chemoreceptors sense this change and triggers the respiratory control centers to increase ventilation. If there is a high controller gain, the change in ventilation may be exaggerated, termed an overshoot. If plant gain is also high, there will be a disproportionately large reduction in PaCO_2_, which will shift to a value below that apneic threshold. This triggers an apneic event that restarts this cycle. In CSR, the CO_2_ reserve, or the difference between eupneic PaCO_2_ and the apnea threshold, is narrowed, making even a modest ventilatory overshoot sufficient to trigger apnea. Breathing will cease until the PaCO_2_ elevates, triggering hyperpnea [125]. This cycle of negative feedback is the principle of loop gain and results in the oscillatory breathing pattern seen in CSR. Healthy individuals have reduced chemoreceptor sensitivity and ventilatory response during NREM sleep, which permits a rise in eupneic CO_2_ levels during sleep that prevents transient decreases in PaCO_2_ from reaching the apneic threshold [126].

The quintessential presentation of CSR is in congestive heart failure (CHF) due to the impact on all three aspects of loop gain. It has been shown that CHF patients have a 2.3–3.5 times greater sensitivity to carbon dioxide compared to controls, resulting in an elevated controller gain and narrowed CO_2_ reserve [127]. The decreased cardiac output and reduced blood flow characteristic of CHF results in chronic carotid body hypoperfusion. This manifestation of delayed mixing gain has been shown to heighten peripheral chemoreceptor sensitivity in rabbits, predisposing to CSR [128]. Central chemoreceptors are hypersensitized in CHF; however, this process is driven by the tonic afferent input from the carotid bodies. Following carotid body denervation in rats, the central chemoreceptor sensitivity normalized, and apnea incidence decreased twofold [129]. Furthermore, because of the increased left ventricular pressures CHF classically presents with pulmonary congestion. This stimulates C-fibers (J-receptors) in the lungs to trigger hyperventilation with subsequent hypocapnia [130]. Due to the already narrowed CO_2_ reserve, a central apnea with CSR is triggered.

### 6.2. Primary (Idiopathic) Central Sleep Apnea

Primary CSA, known as idiopathic CSA, is defined as repeated cessations of breathing with absent respiratory effort that is not associated with an underlying condition. This is a diagnosis of exclusion that is only awarded once other potential causes are ruled out [1].

The pivotal mechanism driving primary CSA is heightened peripheral and central chemoreceptor sensitivity to CO_2_. It has been shown that the hypercapnic ventilatory response in patients with primary CSA is as elevated in patients with CSA due to CHF, despite the former group having no underlying cardiac conditions [131]. Although the mechanisms driving this abnormality are not clearly understood, this demonstrates that it is a primary driver in idiopathic CSA. Arousals accompanied by abrupt hyperventilation may contribute to central apnea by inducing hypocapnia that lowers PaCO_2_ below the apneic threshold [132]. Furthermore, patients with primary CSA have been shown to chronically hyperventilate while both awake and asleep which supports a eupneic PaCO_2_ that lies close to the apneic threshold [133].

Primary CSA of prematurity and primary CSA of infancy are defined as central apneas and/or hypopneas not attributable to another cause occurring before or after 37 weeks conceptual age, respectively [1]. Primary CSA of prematurity is the result of physiological brain stem immaturity. There is a decreased respiratory center output resulting in hyporesponsiveness to changes in CO_2_ [134]. As shown in a recent clinical trial by Mammel et al., preterm infants rely disproportionately on peripheral chemoreceptor input that destabilizes breathing [135]. Additionally, due to the immaturity of carotid body receptors, hypoxia may induce a paradoxical hypoventilation as seen in 65% of premature infants in a prospective observational study by Zannin et al. [136]. This form of CSA is self-limiting and resolves in 98% of infants with maturation to 40 weeks gestational age [137]. Conversely, Primary CSA of infancy is due to a pathological immaturity of the respiratory control systems [138]. This intrinsic dysfunction of the brainstem or chemoreceptors, as opposed to a developmental delay. The mechanisms behind this abnormality are similar to those of primary CSA of prematurity, with an immature brain stem, altered chemoreceptor response, and elevated loop gain playing a critical role [139].

### 6.3. Central Sleep Apnea Due to a Medical Disorder Without Cheyne-Stokes Breathing

CSA without CSR arises from impaired rhythmogenesis or diminished central respiratory drive, producing low loop gain and nonoscillatory central apneas [1]. These disorders lack CSR since the underlying pathology lies within the central pattern generator and lacks the exaggerated chemoreceptor response or circulatory delay.

Cerebrovascular accidents and brainstem neoplasms are additional causes of CSA without CSR [1]. Ischemic injuries to the medullary respiratory neurons can disrupt rhythmogenesis via the pre-BötC and/or chemoreceptor signaling via the RTN [140]. Lesion network mapping studies demonstrated damage that supramedullary brain regions can further contribute to CSA by destabilizing ventilatory control. Injuries to brain networks with connections to the middle cingulate gyrus and bilateral cerebellar posterior lobes were shown to be directly involved in the development of CSA following strokes [141].

Neurodegenerative diseases have been implicated in the pathogenesis of CSA. Multiple system atrophy (MSA) results in the destruction of neurons in the pontomedullary region, including the pre-BötC. The progressive failure of the pattern generator manifests as central apneas [142]. MSA has also been shown to destroy the putative chemosensitive neurons in the ventral medullary surface that drive the hypercapnic ventilatory response [143].

Chiari malformation, which is the herniation of the cerebellar tonsils below the foramen magnum, can cause CSA via compression of the ventrolateral medulla. The resultant compression of the pre-BötC and RTN impairs rhythmogenesis and the sensitivity of chemoreceptors [144]. The mechanisms by which the chemoreceptors are impaired depend on which neuron populations are most affected. There may either be a blunted or excessive response to changes in PaCO_2_, resulting in a heterogeneous presentation [145].

Congenital central hypoventilation syndrome (CCHS) is a disorder caused by polyalanine repeat expansion mutations in the homeobox 2B (PHOX2B) gene. PHOX2B is critical for the development of the RTN, and dysfunctionality results in impairment of central chemoreceptors [146]. A study with mice bearing the most common PHOX2B mutation showed the complete absence of the RTN but normal development of other medullary structures. These mice had no ventilatory response to hypercapnia and died shortly after birth [147]. CCHS additionally presents with hyposensitivity to peripheral chemoreceptors and diminished ventilatory responses to hypoxia as demonstrated by step changes in inspired oxygen [148].

### 6.4. Central Sleep Apnea Due to Medication or Substance

Medication-induced CSA is most commonly seen with opioid use, particularly methadone [149]. Activation of μ-opioid receptors (MOR) in the pre-BötC has been shown to directly inhibit the excitatory pathway of rhythmogenesis in knockout mice studies [150]. The nucleus tractus solitarius (NTS), which receives input from peripheral chemoreceptors, additionally has high concentrations of MORs. The involvement of the NTS is confirmed by a study which demonstrated that injecting an MOR agonist into mice followed by a period of brief hypoxia results in a diminished hypoxic ventilatory response [151]. Causality was established in a case report where discontinuation of opioid use resulted in resolution of the patient’s CSA [152].

GABA-B agonists, such as baclofen, have been shown in vitro to depress ventilation via the same G-protein coupled signaling pathway as opioids in the pre-BötC [153]. Further animal studies showed that baclofen decreased firing rates of respiratory neurons with administration of a GABA-B antagonist reversing that effect [154]. GABA-A and glycine receptor potentiation by ethanol can further contribute. In rats, ethanol administration demonstrated a dose-dependent inhibition in respiratory-related hypoglossal nerve signaling [155]. Benzodiazepines can also contribute to central apneas through similar mechanisms as ethanol [156].

### 6.5. Central Sleep Apnea Due to High Altitude Periodic Breathing

CSA due to high altitudes is a result of ventilatory instability caused by hypobaric hypoxia [1]. At increasing elevations, PaO_2_ decreases which stimulates peripheral chemoreceptors and induces the hypoxia ventilatory response. The subsequent hyperventilation results in a hypocapnic state and predisposes to the PaCO_2_ dropping below the apneic threshold. This hypoxia-induced high loop gain creates an oscillatory crescendo-decrescendo pattern termed periodic breathing [157]. It has been shown that augmented FiCO_2_ eliminated periodic breathing at high altitudes which supports hypocapnia as the main driver [158].

### 6.6. Treatment-Emergent Central Sleep Apnea

Treatment-emergent central sleep apnea (TECSA), also known as complex sleep apnea, is defined as the emergence of central apneas and/or hypopneas following the application of positive airway pressure (PAP) for treatment of obstructive sleep apnea (OSA) [1]. OSA can mask underlying ventilatory instability as the physical obstruction prevents hyperventilation. Once PAP therapy relieves the upper airway obstruction, respirations become more efficient in clearing CO_2_, which lowers PaCO_2_ below the apneic threshold [159]. A study showed that reducing the inspiratory PAP resolves TECSA supports the rationale that elevated tidal volumes driving the PaCO_2_ down was the main driver [160].

TECSA is resolved spontaneously within 8 weeks in 79% of patients. Over time, central chemoreceptor plasticity modulates the chemosensitivity to maintain a stable eupneic CO_2_ and a widened the CO_2_ reserve. Since TECSA often resolves spontaneously, identifying this mechanism prevents unnecessary discontinuation of CPAP or premature escalation to more complex therapies. Very severe OSA and mixed sleep apnea with a central apnea index of ≥5 per hour are risk factors associated with persistent TECSA [161]. Persistent TECSA may indicate patients with persistently elevated loop gain or underlying ventilatory instability that warrant reassessment.

### 6.7. Treatment Recommendations for Central Sleep Apnea

Treatment recommendations for CSA are centered around the use of positive airway pressure (PAP). Due to its wide availability, CPAP is a reasonable consideration for initial CSA treatment despite relatively weak evidence for its efficacy [162]. While CPAP does improve AHI at a clinically meaningful threshold, improvements in excessive sleepiness, central apnea index, cardiac outcomes, hospitalizations, and mortality did not meet the same threshold. Furthermore, treatment response to CPAP must be confirmed with additional polysomnography and review of the device for AHI and central apnea index assessments. Failure of CPAP to effectively treat CSA warrants transitioning to a different mechanism of PAP, such as Bilevel Positive Airway Pressure (BPAP) or Adaptive-Servo Ventilation (ASV).

BPAP differs from CPAP by providing two distinct pressure levels during the breathing cycle, versus a single continuous positive pressure given in CPAP. BPAP delivers a higher inspiratory positive airway pressure and a lower expiratory positive airway pressure, with the difference between the two being pressure support that augments the tidal volume of each breath [163]. According to the American Academy of Sleep Medicine, BPAP with a backup rate is the preferred over BPAP without a backup rate, as this guarantees breaths during the period of absent ventilatory drive [162].

ASV is a bilevel based modality used in the treatment of CSA. In contrast with BPAP, ASV continuously measures airflow or minute ventilation and dynamically varies inspiratory pressure support anticyclically, reducing support when the patient hyperventilates and increasing support when the patient is apnea or hypopneic. This maintains ventilation near a specific target and helps suppress central apnea events and Cheyne-Stokes events more completely than conventional BPAP [164].

For patients with CSA not associated with high loop gain, treatment with PAP is not as effective. This is because these conditions are disorders of brainstem compression and blunted or absent respiratory drive, rather than an oscillatory drive. In CCHS, the primary problem is hypoventilation, and the treatment goal is lifelong assisted ventilation. Treatment ranges from BPAP with a backup rate, for patients needing ventilatory support during sleep or transitioning from tracheostomy to positive pressure ventilation via tracheostomy for those needing 24-h support, especially neonates. Another option for ambulatory patients needing continuous support is diaphragmatic pacing by phrenic nerve stimulation [165]. A 2015 retrospective review evaluated phrenic nerve stimulation in CCHS, and although a small sample size, there was relative success of treatment with 72% of patients successfully ventilated by pacing without tracheostomy [166]. However, it is important to note that tracheal decannulation is not ensured with phrenic nerve stimulation; tracheostomy is often still needed at during sleep to prevent upper-airway obstruction [165,166].

## 7. Obesity Hypoventilation Syndrome

Obesity hypoventilation syndrome (OHS) is defined as the combination of obesity (body mass index ≥ 30 kg/m^2^), sleep disordered breathing, and daytime hypercapnia (PCO_2_ ≥ 45 mmHg at sea level), after ruling out other causes for hypoventilation (Table 4) [167]. OHS is a very severe complication of obesity, with sequelae including heart failure, increased rates of mortality, pulmonary hypertension, and increased rates of hospitalization from acute on chronic respiratory exacerbations [168,169].

Severe obesity is a strong risk factor for the development of obesity hypoventilation syndrome, which is important to recognize given that the prevalence of class III (severe) obesity has nearly doubled in the US from 2000 to the early 2020s [170]. OHS is also commonly associated with OSA, with the prevalence of daytime hypercapnia reaching 24% in patients with OSA and comorbid obesity with BMI ≥ 40 kg/m^2^) [170]. Furthermore, approximately 90% of patients with OHS have comorbid OSA, with around 70% having severe OSA with AHI ≥ 30. In patients with OSA, a higher AHI is an independent predictor of OHS even after adjustment for BMI and social factors [171,172]. The clinical complications of OHS involve serious comorbidities and underscore the importance proper diagnosis and management.

### 7.1. Impaired Respiratory Mechanics

Obesity significantly impacts respiratory muscle function and pulmonary mechanics, especially in severe obesity. Patients with OHS are consistently breathing at lower lung volumes. Functional residual capacity (FRC) is markedly reduced in obesity, with some reductions being so great that they approach residual volume (RV). Since breathing occurs at a low FRC, it lies in the less compliant portion of the pressure volume curve. An increase in effort is needed to overcome the increased elasticity of the lungs, which results in obese individuals doing more overall respiratory work to maintain proper ventilation [173].

In OHS, total respiratory system compliance is decreased, driven primarily by decreased lung compliance with a smaller contribution from decreased chest wall compliance, with an increase in airway resistance. Reduced airway diameter from increased upper airway adipose tissue contributes to increased airway resistance [174,175]. Additionally, at the low expiratory reserve volume in OHS, there is small airway collapse and air trapping. The result is tidal expiratory flow limitation which can reach its maximum rate at rest, meaning the patient is unable to increase expiratory flow regardless of additional respiratory effort [173]. Tidal expiratory flow limitation leads to the development of an intrinsic positive end expiratory pressure (PEEP) [176]. This occurs because when there is incomplete expiration, an elastic recoil pressure remains in the respiratory system that imposes a threshold load on the inspiratory muscles that must be overcome before inspiratory flow can be achieved. The result is in increased work of breathing in these patients [173].

Respiratory function in obese individuals worsens in the supine position and during sleep. Not all obese individuals have an intrinsic PEEP while upright and may only develop it while supine. Although a small sample size, Pankow et al. reported a threefold increase in PEEP in obese individuals in the supine versus upright position [177]. The increased adipose tissue around the ribcage and abdomen and in the visceral cavity increases intra-abdominal and pleural pressures, subsequently reducing downward motion of the diaphragm [176,178]. The cephalic movement of the diaphragm likely decreases total lung capacity because it limits how much the lung can expand on inspiration and further reduces FRC [178]. Since FRC is already notably decreased in obesity, further reductions lead to respiration at even lower lung volumes that are more prone to collapse, leading to increased air trapping, tidal expiratory flow limitation, and intrinsic PEEP [177,178].

### 7.2. Respiratory Muscles

Obesity changes the shape of the diaphragm and its ability to generate pressure, as it is elevated in obese versus non-obese individuals and limits its ability to move downwards. Respiratory muscle function seems to be more impaired with increasing severity of obesity, and this may be due to a myopathy or an increased burden on the diaphragm but obesity. Animal studies using obese mice have demonstrated intrinsic diaphragmatic changes, such as adipose tissue and collagen deposition that increases with contractile dysfunction [177]. Thrombospondin-1, a circulating adipokine that is increased in obesity, is thought to mediate this process as obese thrombospondin-1 knockout mice were protected from the diaphragm changes despite similar weight gain as thrombospondin-1 producing mice [179]. These findings have been preliminarily evaluated in humans via ultrasound and MRI. The diaphragm thickening fraction is a surrogate for contractile force and correlates with respiratory muscle function, meaning the diaphragm thickens more with increased force of contraction. Diaphragmatic thickening fraction is decreased in obese individuals compared to non-obese individuals in both quiet and forced breathing [180,181,182]. In 2023, Xiang et al. measured the proton density fat fraction to be significantly higher in the diaphragm of obese individuals, with immunostaining confirming the high proportion of lipid droplets in these diaphragms [180]. While the mechanical forces causing cephalad displacement of the diaphragm are more well established, intrinsic changes in the muscle may also be associated with impaired contractile ability.

The pressure generating capacity of the diaphragm can be numerically characterized by the maximum inspiratory pressure (MIP) and maximum expiratory pressure (MEP). MIPs and MEPs are generally decreased in patients with obesity, but the results were not significantly lower than normal limits to cause alveolar hypoventilation alone [175,183,184]. Respiratory muscle endurance is significantly decreased in OHS. After bariatric surgery with a BMI reduction of 10 kg/m^2^, inspiratory (MIP) and expiratory (MEP) muscle strength increased by 21% and 22% respectively, with respiratory muscle endurance also increasing by 13%. FVC and FEV1 only increased by 9% and 3% respectively. Taken together, this study implies a direct effect of weight reduction on improved respiratory function [185].

### 7.3. Blunted Ventilatory Drive and Leptin Resistance

Leptin plays a critical role in the pathogenesis of OHS due to development of resistance to respiratory effects of leptin, resulting in a blunted hypercapnic respiratory response and decreased airway patency. Leptin is an adipocyte produced hormone that is responsible for increasing metabolic rate, stimulating breathing, and increasing airway patency. The central metabolic and respiratory effects of leptin are transmitted by action on the Ob-Rb or LEPr3 isoform of the leptin receptor [186]. Once the receptor is bound by leptin, JAK2 is activated which phosphorylates STAT3, a transcription factor that promotes POMC transcription [186,187]. This signaling pathway is located in the arcuate nucleus and dorsomedial hypothalamus, as well as the nucleus tractus solitarius in the medulla [186,188,189]. Obesity caused by increased positive energy balance, the most common form of obesity in humans, shows an increase in leptin out of proportion to adipose tissue mass. In non-obese individuals, leptin tends to circulate at 5–10 ng/mL, but can increase to 98 ng/mL in obese individuals with body fat mass reaching over 30% [190]. Despite elevated leptin levels, these patients are still hyperphagic and, notable for the pathogenesis of OHS, resistant to the respiratory stimulating effects of leptin [186].

Selective central leptin resistance is found in OHS and is described as attenuated central leptin effects even with hyperleptinemia [186,191]. The reasoning behind this is multifactorial. First, increased leptin levels can cause impaired transport across the blood brain barrier [192]. In obese mice, the rate of leptin transport across the blood brain barrier is about two-thirds that of non-obese mice [193]. Secondly, impairments in the LEPR3 signaling pathway are hypothesized to cause leptin resistance. Decreased trafficking or increased endocytosis of plasma membrane LERP3, reduced phosphorylation of STAT3, and upregulation of the suppressor of cytokine signaling 3 and protein tyrosine phosphatase 1B cause LEPR3 dysfunction [186,194].

### 7.4. Leptin’s Effect on the Respiratory Control Centers

#### 7.4.1. Leptin and the Nucleus Tractus Solitarius (NTS)

The NTS, located in the dorsolateral medulla, plays a crucial role in processing afferent information from peripheral chemoreceptors and respiratory modulation. LEPR3 signaling pathways have been found in NTS neurons [189]. Direct administration of leptin to the NTS can augment breathing during sleep. Hypercapnic respiratory response and activity of inspiratory muscles increased with one administration of leptin to the NTS in anesthetized rats [194,195]. Another study was able to directly stimulate LEPR positive neurons in the NTS, which increased phrenic nerve burst amplitude. Additionally, the LEPR positive neurons project to the inspiratory premotor region and dorsomedial hypothalamus, both of which are vital in the control of breathing [196,197]. However, when LEPR positive neurons of the NTS were activated in diet induced obese mice during sleep, there was no significant stimulation of breathing or upper airway muscles [198]. This suggests that although leptin seems to stimulate breathing via the NTS, it may not have a prolonged, strong enough effect to play a therapeutic role in OHS.

#### 7.4.2. Leptin and the Retrotrapezoid Nucleus

The retrotrapezoid nucleus is very sensitive to hypercapnia and is the major central nervous site where ventilation is regulated by pH and CO_2_ [186]. GPR4 and TASK-2 detect rising H^+^/CO_2_, which causes downstream stimulation of respiratory pattern generators to increase ventilation [199]. Leptin has also been implicated in RTN activation via the JAK2/STAT3 pathway. Wei et al. examined the effects of obesity on RTN activation in a diet induced obesity mouse model. When exposed to room air, diet-induced obese mice had baseline hypoventilation with rapid and shallow breathing, with an impaired hypercapnic respiratory response upon exposure to CO_2_. The number of CO_2_ activated neurons as well as the expression of TASK-2 decreased, hindering downstream stimulation of ventilation. Phosphorylated STAT3 was also downregulated, with an upregulation in SOCS3, a feedback inhibitor of leptin signaling [200]. When leptin is directly administered to the rostral ventrolateral medulla of obese mice, however, there is an increase in baseline ventilation, minute ventilation, tidal volume, and hypercapnic ventilatory response [189]. Combined, these results demonstrate that obesity may contribute to dysregulated leptin signaling, but that targeted leptin administration in the RTN can alleviate the effects of obesity on ventilation.

#### 7.4.3. Leptin and the Dorsomedial Hypothalamus

Leptin may regulate the control of the upper airway and breathing through effects on the dorsomedial hypothalamus. This was demonstrated by Yao et al. in a study that administered leptin to the lateral ventricle with a resulting increase in inspiratory flow, minute ventilation, and tidal volume [189]. A subsequent study found that intracerebral leptin in the dorsomedial hypothalamus treated OHS in leptin deficient obese mice through increased ventilation [201]. Interestingly, leptin signaling neurons in the dorsomedial hypothalamus project to the hypoglossal nucleus [189]. The genioglossus is the prominent upper airway dilator and is controlled by the hypoglossal nerve. Together, this suggests that leptin may contribute to hypoglossal activation and subsequent maintenance of upper airway patency during sleep.

Thus far, data from animal studies demonstrates that leptin and the LEPR signaling pathway are integral in the pathogenesis of OHS. This provides pre-clinical evidence that leptin agonists could be a therapeutic modality for treatment of OHS in humans. However, this is still in the very early stages of development. Further research would be needed to clarify which, if any, leptin targets would be therapeutically actionable in humans, as well as translational and clinical studies to assess OHS response to leptin agonists in humans.

### 7.5. Treatment Recommendations for Obesity Hypoventilation Syndrome

Positive airway pressure (PAP) therapy remains the cornerstone treatment of OSA according to the American College of Chest Physicians and the American Journal of Respiratory and Critical Care Medicine [202,203]. OHS symptoms, both awake and asleep, improve subjectively and objectively with use of PAP. In two randomized controlled trials from 2015 and 2016, improvements in PaO_2_ and PaCO_2_ were seen in patients treated with PAP [204,205]. Regarding sleep quality, the microarousal index on polysomnography improved with PAP treatment, as well as better scores on the Functional Outcome of Sleep questionnaire and Epworth Sleepiness Scale [202]. The patients most likely to experience the aforementioned benefits of PAP treatment are patients with symptomatic OHS with comorbidities and those with chronic respiratory failure after an episode of acute respiratory failure. Currently, there is not enough evidence to recommend specific monitoring parameters, but at a minimum, continuous pulse ox monitoring overnight is suggested, as well as regular assessment of adherence to and proper usage of PAP [202].

PAP can be delivered via CPAP or non-invasive ventilation (NIV). Observational studies and RCTs have demonstrated improvements in objective measures, including PaCO_2_, PaO_2_, and the need for awake oxygen supplementation, and daytime sleepiness, as assessed by the Epworth Sleepiness Scale, with both CPAP and NIV. However, since greater than 70% of patients with OHS have concomitant OSA, CPAP is suggested as the initial treatment given its known benefits in treating OSA [202].

## 8. Clinical Implications

Diagnosing Sleep Disordered Breathing by cut-off values on a polysomnography would be an oversimplification. Obstructive Sleep Apnea, as an example can be further understood through the lens of the patient symptoms, polysomnographic parameters, and underlying pathophysiology. There is utility in clinical practice to examine the symptoms to identify the phenotype of OSA, as it can impact the management and allow monitoring of those symptoms with intervention. With the advent of machine learning, we face the challenge of capturing clinical data and deciding which inputs to use to enhance our understanding of clusters of OSA and their relationship to co-morbidities and survival. Ma and others examined a number of parameters such as sleep architecture, respiratory events, oxygen desaturation, and limb movements during sleep to refine six clusters of OSA. Overall, the lowest risk clusters were C1, with low AHI and minimal hypoxia, and C2, notable for moderate AHI and mild sleep disruption [206]. In contrast, other clusters that had a mix of severe hypoxia, sleep fragmentation, and high AHI had the highest risk for hypertension and cardiovascular disease. The data that we capture through polysomnography, assessments, and clinical practice need to be standardized and accessible to allow for the diagnosis not just of obstructive sleep apnea, but of the cluster, phenotype, and/or endotype of obstructive sleep apnea. Machine learning is limited due to the wide scattering of data throughout the medical chart with the lack of a framework to be useful for risk stratification, precision medicine, and correlation with health outcomes.

## 9. Conclusions

Sleep disordered breathing represents a complex array of conditions with unique and variable mechanisms driving the disease process. The heterogeneity of pathophysiology presents challenges in diagnosing, classifying, and treating these series of diseases. The AHI as measured from polysomnography is the primary metric used for making a diagnosis and determining disease severity; however, it is an imperfect tool that frequently oversimplifies the diversity of sleep apnea. A recent review highlighted these shortcomings and urged for better characterization of these disorders by utilizing more of metrics provided by the polysomnography [207]. Furthermore, it is of paramount importance to clinically correlate these findings as the presentations of these disorders are widely varied.

Understanding the pathophysiology of these disorders is fundamental in successful treatment as well as mitigation of the long-term consequences. When taken in combination with a diversified approach to classification, there is potential to develop tailored treatment plans that align with the pathophysiologic mechanisms driving a patient’s disease. Treatment currently focuses on decreasing AHI, with positive airway pressure being the primary treatment and weight loss [208]. Other treatment options that focus on maintaining airway patency include oral appliances, surgical intervention, and hypoglossal nerve stimulation. Pharmacotherapy for both obstructive and central sleep apnea is available and targets the underlying disease pathways. Overall, an understanding of the pathophysiological mechanisms driving sleep disordered breathing is critical for personalization of treatment to most effectively reduce the consequences of sleep disordered breathing.

## Figures and Tables

**Table 1:** Primary snoring and upper airway resistance syndrome.

Core mechanism	Increased upper airway resistance without significant oxygen desaturation (UARS) or obstructive apneas (primary snoring); increased respiratory effort and sleep fragmentation
Clinical consequences	Snoring, daytime fatigue, reduced quality of life; UARS may progress to OSA; may be associated with adverse cardiovascular changes
Diagnostic considerations	PSG may demonstrate respiratory effort-related arousals (RERAs) despite low AHI; diagnosis often requires clinical correlation and exclusion of OSA
Treatment implications	Lifestyle modification, weight management, positional therapy, oral appliances, and CPAP in symptomatic UARS; treatment individualized based on symptoms and disease severity

**Table 2:** Obstructive sleep apnea.

Core mechanism	Recurrent upper airway collapse during sleep causing intermittent hypoxia, hypercapnia, sleep fragmentation, and increased sympathetic activation
Clinical consequences	Excessive daytime sleepiness, impaired cognition, cardiovascular and metabolic disease, increased accident risk
Diagnostic considerations	PSG demonstrating obstructive apneas/hypopneas, assessment of symptom burden and severity
Treatment implications	CPAP, weight loss, oral appliances, positional therapy, upper airway surgery, hypoglossal nerve stimulation

**Table 3:** Central sleep apnea.

Core mechanism	Reduced or absent central respiratory drive leading to diminished ventilatory effort; often associated with heart failure, neurologic disease, opioid use, or high altitude
Clinical consequences	Sleep fragmentation, nocturnal hypoxemia, worsening heart failure symptoms, increased mortality
Diagnostic considerations	PSG showing absent respiratory effort during apneic events; evaluate for underlying cardiac, neurologic, medication-related, or metabolic causes
Treatment implications	Treat underlying cause; CPAP or bilevel PAP; adaptive servo-ventilation when appropriate; supplemental oxygen or phrenic nerve stimulation

**Table 4:** Obesity hypoventilation syndrome.

Core mechanism	Obesity-related chronic alveolar hypoventilation due to impaired respiratory mechanics, blunted ventilatory drive, and sleep-disordered breathing
Clinical consequences	Chronic hypercapnia, hypoxemia, pulmonary hypertension, right heart failure, increased hospitalization and mortality
Diagnostic considerations	BMI ≥ 30 kg/m^2^ with awake hypercapnia (PaCO_2_ ≥ 45 mmHg) after excluding other causes; PSG often demonstrates coexisting OSA
Treatment implications	Weight loss (including bariatric surgery), PAP therapy (CPAP if OSA predominates, NIV for persistent hypoventilation), management of cardiometabolic comorbidities

## Data Availability

No new data were generated or analyzed in this study. All data is publicly available on PubMed (https://pubmed.ncbi.nlm.nih.gov/) accessed on 1 March 2026.

## Abbreviations

The following abbreviations are used in this manuscript:

AHI	Apnea-hypopnea index
ASV	Adaptive-Servo Ventilation
BMI	Body mass index
BPAP	Bilevel Positive Airway Pressure
CCHS	Congenital central hypoventilation syndrome
CHF	Congestive heart failure
CIH	Chronic intermittent hypoxemia
CPAP	Continuous positive airway pressure
CSA	Central sleep apnea
CSR	Cheyne-Stokes respirations
EEG	Electroencephalogram
FRC	Functional residual capacity
MASLD	Metabolic dysfunction-associated steatotic liver disease
MEP	Maximum expiratory pressure
MIP	Maximum inspiratory pressure
MOR	μ-opioid receptors
MSA	Multiple system atrophy
NTS	Nucleus tractus solitarius
NREM	Non-rapid eye movement
OHS	Obesity hypoventilation syndrome
OSA	Obstructive sleep apnea
PAP	Positive airway pressure
Pcrit	Pharyngeal critical closing pressure
PEEP	Positive end expiratory pressure
PHOX2B	Paired-like homeobox 2B
PLMS	Periodic limb movements of sleep
pre-BötC	pre-Bötzinger complex
PSG	Polysomnography
REM	Rapid eye movement
RERA	Respiratory effort related arousals
RTN	Retrotrapezoid nucleus
RV	Residual volume
SDB	Sleep-disordered breathing
SCN	Suprachiasmatic nucleus
SNS	Sympathetic nervous system
SWA	Slow wave activity
T2DM	Type 2 diabetes mellitus
TECSA	Treatment-emergent central sleep apnea
UARS	Upper airway resistance syndrome
